# Posterior capsular radial sign: a novel method to confirm anterior vitreous cortex resection in phacovitrectomy

**DOI:** 10.1186/s12886-024-03474-x

**Published:** 2024-05-31

**Authors:** Shen Qu, Qi Zhou, Yu-Ting Shao, Ming-Yue Lin, Jia-Qi Shen, Guo-Zhen Niu, Wen-Ting Han, Li Zhang, Yan-Long Bi

**Affiliations:** Department of Ophthalmology, Tongji Hospital, School of Medicine, Tongji University, Shanghai, 200065 China

**Keywords:** Threadiness corrugation, Radial folds, Anterior vitreous cortex, Silicone oil

## Abstract

**Background:**

The main purpose of this paper is to introduce a method that can accurately locate the posterior capsule of the lens to facilitate a relatively complete resection of the anterior vitreous body.

**Methods:**

A total of 51 patients in the experimental group and control group were enrolled in this study. Phacoemulsification combined with vitrectomy was performed in all cases. After the cataract procedure was completed in the control group, the surgeon performed a conventional anterior vitrectomy with the operative eye. In the experimental group, anterior vitrectomy was performed according to the threadiness corrugation of the posterior capsule of the lens. During the operation, with the help of triamcinolone, two surgeons confirmed the resection of the anterior vitreous cortex; the best corrected visual acuity and intraocular pressure of all patients were recorded at 1 week, 1 month and 3 months after surgery.

**Results:**

Fifty patients underwent phacoemulsification combined with vitrectomy, except one patient in the experimental group who was lost to follow-up. After surgery, no significant complications were observed in all patients except two patients in the control group with temporary increases in intraocular pressure. There was no significant difference in preoperative visual acuity between the two groups (*t* = 0.83, *P* = 0.25). Both groups had varying degrees of improvement in best corrected visual acuity at 1 week, 1 month and 3 months after surgery. Moreover, there was no significant difference in BCVA between the two groups at the three follow-up time points (*t*=-1.15, -1.65, -1.09, *P* = 0.53, 0.21, 0.23). After surgery, no significant complications were observed in all patients except two patients in the control group with temporary increases in intraocular pressure. Incomplete resection of the anterior vitreous cortex was observed in 2 patients in each group, but there was no significant difference (χ^2^ *= 7.81, P > 0.05*).

**Conclusion:**

In the process of cataract surgery combined with vitrectomy, thready corrugation appears in the posterior capsule of the lens and is an important sign of its localization. Anterior vitrectomy can be accomplished safely and effectively with the help of thread-like corrugation, and the surgical effect is almost the same as that of traditional surgery. Especially suitable for beginners in vitreous surgery.

## Background

Minimally invasive vitrectomy combined with phacoemulsification and intraocular lens implantation has been widely used in the treatment of patients with cataract and vitreoretinal disease [1–5]. Under normal conditions, when the intraocular lens was implanted, vitreous surgery is the next step. Anatomically, there is no definition that divides the vitreous body into anterior or posterior segments. Clinically, for some diseases such as posterior capsular opacification that cannot be effectively treated by *Nd: YAG* laser, dislocation of the lens, malignant glaucoma, etc. Only part of the vitreous body behind the posterior capsule of the lens needs to be removed, which is called “anterior vitrectomy”. In combined operation, removing as many anterior vitreous body as possible can effectively prevent intraocular proliferation [6]. This has important implications for some diseases, such as proliferative diabetic retinopathy (PDR), giant hole retinal detachment and proliferative vitreoretinopathy (PVR), especially after silicone oil tamponade surgery [7–9]. It is well known that the emulsification and migration of silicone oil can cause many postoperative complications, including secondary glaucoma, corneal degeneration or decompensation [7, 10, 11], and even the introduction of silicone oil into the skull has been reported [12]. Many clinical studies have suggested that silicone oil should be removed approximately 3 to 6 months after vitrectomy [13–16]. The residual silicone oil droplets attached to the posterior lens capsule after silicone oil removal is one of the reasons to accelerate the opacity and affect the postoperative visual acuity [17–20]. Reducing the residual silicone oil droplets under the posterior capsule of the lens can effectively delay its opacity [21]. This requires the surgeon to remove the anterior vitreous cortex as completely as possible during the procedure. For novice surgeons, the implantation of intraocular lenses and the polishing of the posterior capsule make it difficult to locate the position of the anterior vitreous body. Through long-term intraoperative observation, we attempt to share a method that can achieve relatively complete excision of the anterior vitreous body by localization of the posterior capsule of the lens in this article.

## Methods

### Patients

This retrospective study included 51 patients who had undergone phacoemulsification and intraocular lens implantation combined with vitrectomy in the Department of Ophthalmology, *Tongji* Hospital affiliated to *Tongji* University. The whole study was approved by the Ethics Committee of *Tongji* Hospital and conformed to the *Helsinki Declaration*. All patients were informed of the purpose of the study and voluntarily signed informed consent before surgery. Both groups were diagnosed with cataracts and various fundus diseases, including vitreous hemorrhage, retinal detachment, and proliferative diabetic retinopathy. History of cataract surgery, intravitreal injection of anti-VEGF drugs, trabeculectomy and drainage valve implantation were not included in this study. Before the operation, patients are routinely given general examinations such as electrocardiogram, routine blood examination, coagulation function, liver and kidney function, fasting plasma glucose, blood pressure, etc. None of the patients had a systemic disease that made them unable to tolerate surgery. Relevant ophthalmic testing include best correlated visual acuity (BCVA), intraocular pressure (IOP), slit lamp biomicroscopy, funduscopy, ocular *B*-ultrasound, etc. The surgical procedures were performed by the same ophthalmologist using the *25-gauge* vitrectomy system (constellation surgical system, Alcon Surgical Inc., Fort Worth, TX). After completing the steps of cataract surgery, 26 patients in the control group underwent routine anterior vitrectomy and were observed directly under a microscope. The experimental group of 25 patients underwent the same procedure. Unlike the control group, threadiness corrugation of the posterior capsule of the lens was used as a marker for location anterior vitrectomy during the operation.

### Surgery

**Fig. 1:**
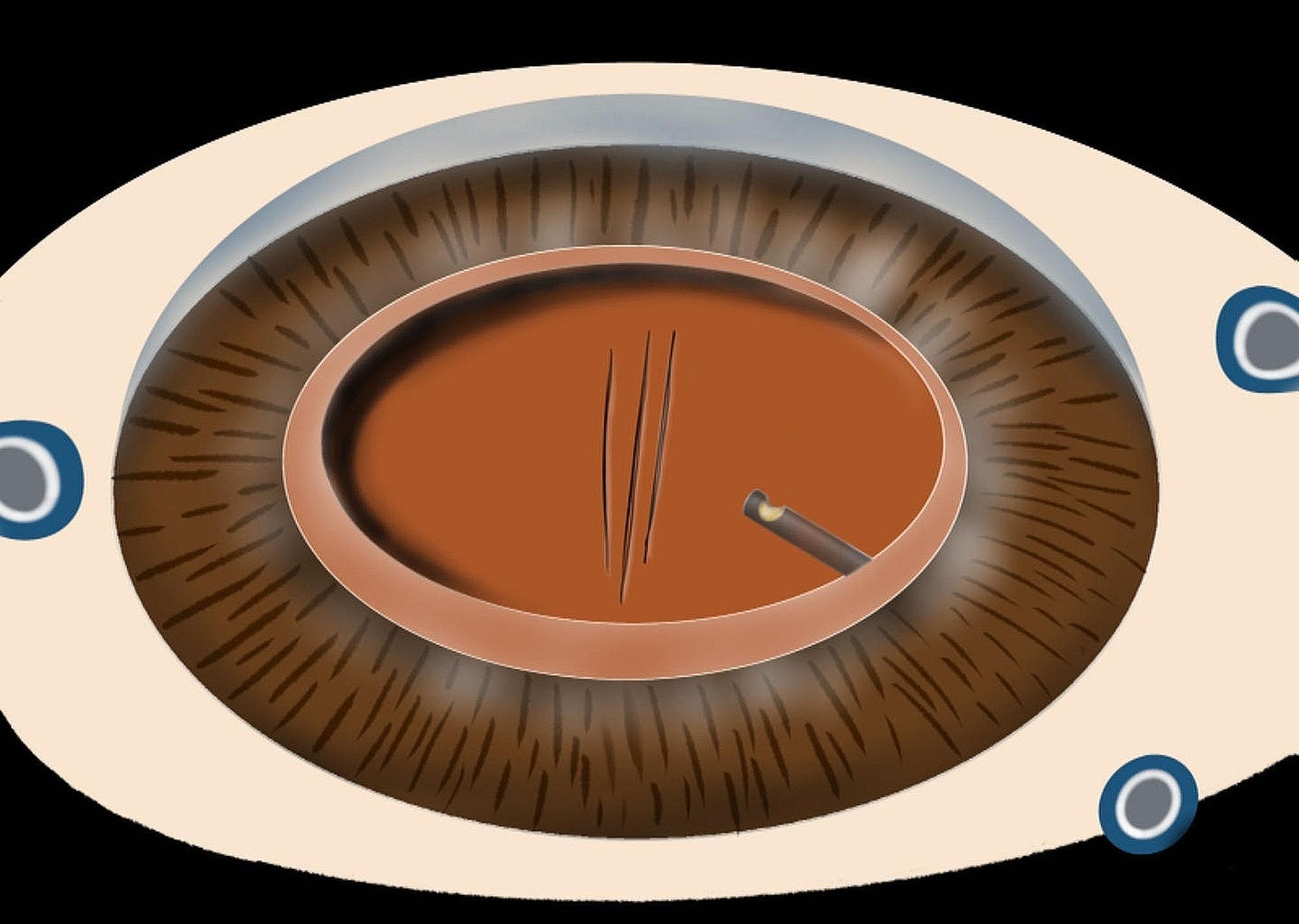
Shows that the “threadiness corrugation” appears in the posterior capsule of the lens after implantation of an IOL

**Fig. 2:**
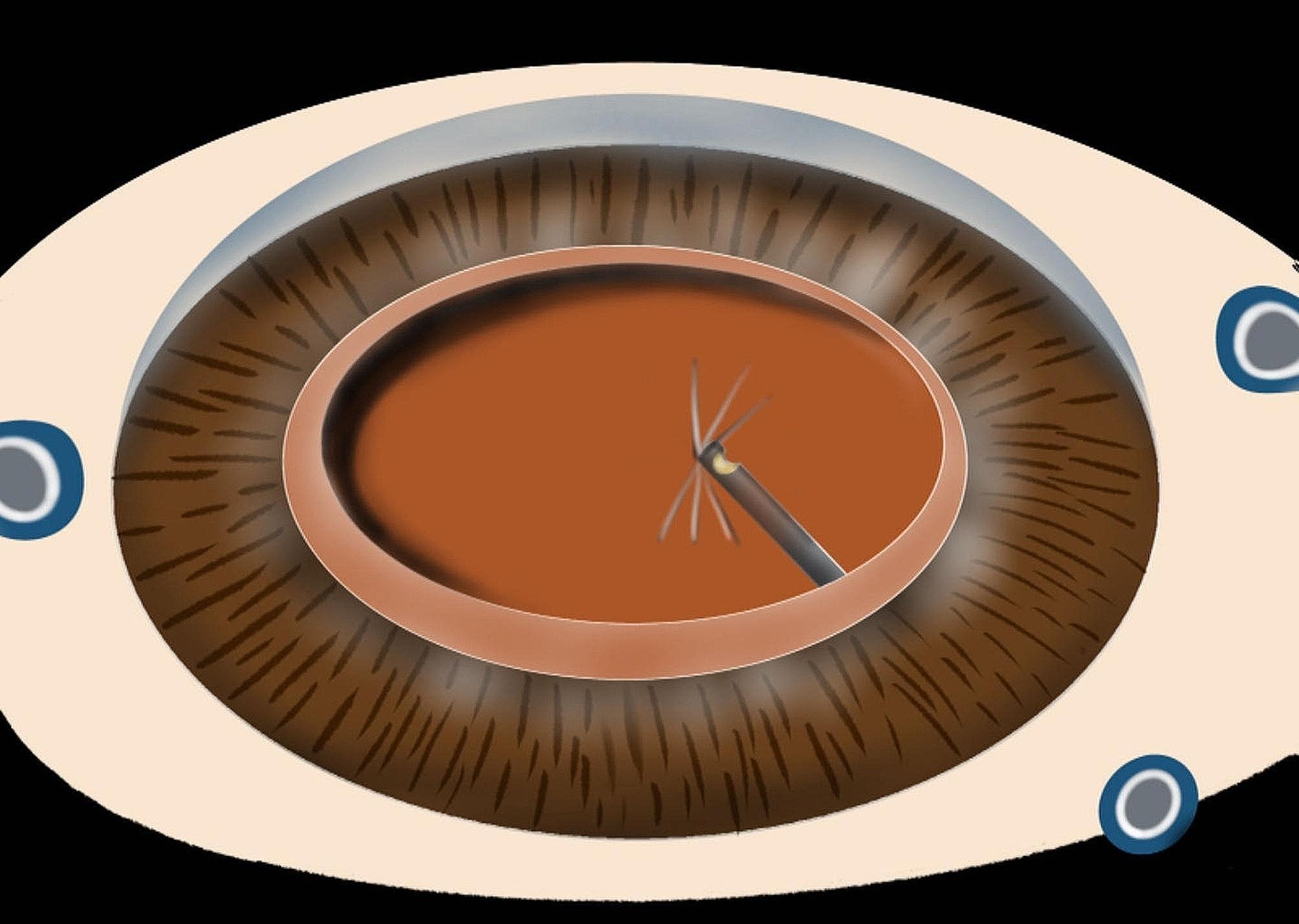
Shows the “radial folds” of the posterior capsule of the lens when the anterior segment of the vitreous body behind the posterior capsule is excised

All procedures were performed by the same experienced ophthalmologist, and the whole operation was performed under retrobulbar anesthesia. The specific steps were as follows: a corneal incision and lateral incision were made at the 11 and 3 o’clock positions above the corneal limbus. Appropriate amounts of *medical hyaluronan gel* (Bausch & Lomb Inc., Shangdong, China) were injected into the anterior chamber. After multidirectional divide and conquer, the nuclei lentis and cortex were removed by phacoemulsification after continuous circular capsulorheixis. After polishing the posterior capsule, *medical hyaluronan gel* was injected into the anterior chamber again. The intraocular lens of the corresponding diopter was implanted in the capsulalentis. It is important to emphasize here that the *medical hyaluronan gel* was aspirated and then reinjected into the anterior chamber, and the corneal incision was closed with *10 − 0* nylon suture. Then, *25G* minimally invasive vitrectomy was performed. The infusion pressure is 25 mmHg, the cutting rate is 5000 cpm and the vacuum power is 300 mmHg. We can see that a “*threadiness corrugation”* (Fig. 1) appears in the central region of the posterior capsule, which is an important marker for positioning the posterior capsule of the lens. This allows the surgeon to carefully remove the anterior vitreous body behind the lens, avoiding accidental breakage to the posterior capsule of the lens. After the anterior vitrectomy was complete, the operation mode was changed to negative pressure suction, and the cutter head was attached to the posterior capsule to verify whether there was any vitrectomy body remaining below the posterior capsule. If “*radial folds”* (Fig. 2) appear at this time, the anterior vitreous body has been completely removed. Conversely, if the whole posterior capsule is in a flapping state, there is still a remnant of the vitreous cortex. After anterior vitrectomy, injecting an appropriate amount of triamcinolone into the vitreous cavity can help complete the process of posterior vitreous detachment, and the surgeon can observe whether the vitreous anterior cortex has been excised.

### Statistical analysis

Data treating in this study was analyzed using *SPSS 22.0* statistical software (SPSS Inc., Chicago, IL, USA). All data conformed to a normal distribution and are expressed as the *mean ± standard* deviation (*M ± SD*). *T test* was used for comparison of measurement data between groups. *Chi-square* tests were used to compare counting data between groups. A *P* value less than *0.05* indicated a statistically significant difference.

## Results

### Characteristics

A total of 25 patients were included in the experimental group, including 14 males and 11 females, and 1 male patient was lost to follow-up. The average age of the patients in the experimental group was 57.17 ± 5.16 (range 59–78) years. The control group completed follow-up of 26 patients, including 12 males and 14 females. The average age of the patients in the control group was 59.22 ± 4.48 (range 61–80) years. All operations in the two groups were successfully completed, and no serious postoperative complications were found. The intraocular lens was implanted in the lens capsule, and there was no rupture of the posterior capsule of the lens in any of the patients. In the control group, there were 2 patients with a transient increase in intraocular pressure (IOP) in the short term after the operation, and the intraocular pressure decreased to normal levels after brinzolamide eye drops (ALCON-COUVERUR n.v. Belgium) or Carteolol Hydrochloride Eye Drops (China Otsuka Pharmaceutical Co., Ltd.) treatment. There was no significant difference in age (*P* = 0.765), gender (*P* = 0.612), plasma glucose (*P* = 0.422), HBA1c (*P* = 0.185), hypertension (*P* = 0.741), best corrected visual acuity (*P* = 0.283), or intraocular pressure (*P* = 0.456) between the two groups. (Table 1)

**Table 1:** Clinical characteristics of patients in the two groups

	Control group	Experiment group	T value	*P* value
Number of eyes	24	26	-0.11	0.60
Gender				
Male	13	12	0.43	0.91
Female	11	14	0.69	0.41
Age	59.22 ± 4.48	57.17 ± 5.16	0.67	0.76
Plasma glucose	7.49 ± 0.71	7.27 ± 0.61	0.96	0.42
HBA1c	5.50 ± 1.13	6.66 ± 0.76	-3.51	0.18
Hypertension	10	11	0.11	0.74
BCVA (logMAR)	0.93 ± 0.73	0.90 ± 0.10	0.83	0.25
IOP (mmHg)	13.73 ± 2.13	13.53 ± 1.81	0.23	0.45

### BCVA and IOP

The best correlated visual acuity and intraocular pressure were recorded in all patients before surgery and at 1 week, 1 month and 3 months after surgery. Before surgery, the BCVA of the two groups was 0.93 ± 0.73 and 0.90 ± 0.10, with no statistically significant difference (*t* = 0.83, *P* = 0.25). All patients in the two groups achieved varying degrees of improvement in BCVA after the operation. There was no significant difference in visual acuity at different times after surgery between the two groups. Similarly, there was no significant difference in IOP between the two groups at each time point after surgery. (Table 2)

**Table 2:** BCVA and IOP after surgery

	Control group	Experiment group	T value	*P* value
BCVA (LogMAR)				
Baseline	0.93 ± 0.73	0.90 ± 0.10	0.83	0.25
1 week	0.62 ± 0.18	0.69 ± 0.14	-1.15	0.53
1 month	0.50 ± 0.08	0.56 ± 0.12	-1.65	0.21
3 month	0.48 ± 0.08	0.52 ± 0.61	-1.09	0.23
IOP (mmHg)				
Baseline	13.73 ± 2.13	13.53 ± 1.81	0.23	0.45
1 week	14.02 ± 3.32	15.22 ± 1.96	0.19	0.36
1 month	12.77 ± 2.24	12.89 ± 1.92	0.31	0.22
3 month	13.12 ± 1.77	12.75 ± 1.55	0.26	0.19

### Verification of triamcinolone

**Fig. 3:**
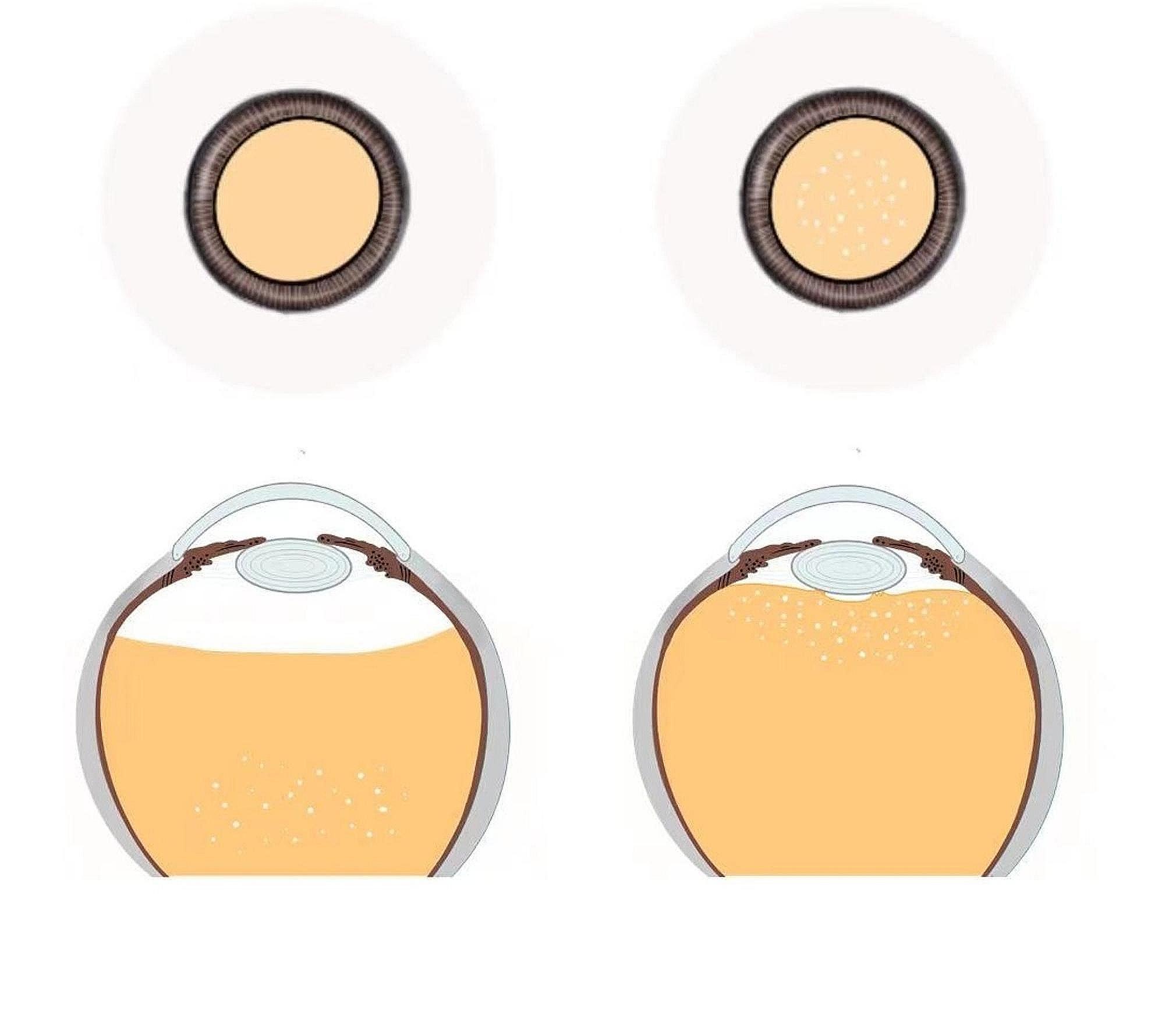
Shows the dispersion of dexamethasone in the vitreous cavity from the observation of two doctors

In view of the fact that there is still no effective method to quantitatively analyze the vitreous body, this study used triamcinolone to artificially observe the resection of the vitreous anterior cortex. After the surgeon excises the anterior segment of the vitreous body under the posterior capsule of the lens, triamcinolone acetonide is injected into the vitreous cavity. Both the surgeon and the assistant confirm the dispersion of triamcinolone in the vitreous cavity: if it settles completely below the vitreous cavity, the clearance of the vitreous anterior cortex is relatively complete; if the clearance of the anterior vitreous cortex is not complete, triamcinolone is still visible in the vicinity of the posterior capsule of the lens (Fig. 3). In this study, we observed that 2 patients in each group still had a small remnant of the anterior vitreous cortex (Table 3). There was no significant difference (χ^2^ *= 7.81, P > 0.05*).

**Table 3:** The number of excisions of the vitreous anterior cortex

Group	Results		Total
	(+)	(-)	
Experiment	24	2	26
Control	22	2	24
Total	26	4	50

“+” means that the anterior vitreous cortex has been removed completely, and “-” means that it remains.

## Discussion

Recent improvements in vitrectomy techniques have contributed to expanding the role of pars plana vitrectomy (PPV) in the management of certain vitreoretinal diseases. In consideration of the complexity of vitreoretinal diseases and the unexpected conditions that may arise during surgery. Novice surgeons need sufficient time to achieve an acceptable success rate [22]. Viola [23] and colleagues’ study showed that beginners needed more than twice the time for the procedure compared to experienced surgeons. Resection of the anterior vitreous cortex is particularly important during combined anterior and posterior segment surgery, especially for cases requiring intraoperative silicone oil tamponade. Silicone oil remnants is a common postoperative complication of silicone oil removal. Even after repeated intraoperative gas-liquid exchange, it is difficult to avoid the hiding of silicone oil or some impurities behind the posterior capsule of the lens. Residual and emulsified silicone oil droplets can migrate to the anterior chamber, leading to a series of postoperative complications, including secondary glaucoma, after-cataract and corneal decompensation. Studies [24, 25] have shown that intraoperative removal of the anterior vitreous cortex as much as possible can reduce the adhesion and even migration of silicone oil droplets. As mentioned above, vitrectomy is a complex ophthalmologic operation with a relatively long learning curve. Even with the help of the microscope, complete removal of the anterior vitreous cortex is not easy for novice surgeons. In this study, we hope to use the threadiness corrugation that appears in the posterior capsule of the lens as a marker to locate the anterior vitrectomy.

The principle of this method is relatively clear: during the phacoemulsification process, the entire posterior capsule of the lens becomes slack and tends to drift forward after the nucleus and cortex are aspirated. It is like pulling something heavy out of a “plastic bag”. The bag is wrinkled due to lack of support. At this time, “threadiness corrugation” can be observed in the posterior capsule of the lens. The surgeon can use this to locate the posterior capsule of the lens to remove the anterior vitreous cortex. It should be noted here that *medical hyaluronan gel* should be thoroughly aspirated. Otherwise, due to the supporting effect of *medical hyaluronan* gel, the posterior capsule of the lens will be distended. In addition, some surgeons have a habit of polishing the anterior capsule. These procedures make the posterior lens capsule difficult to distinguish and thus easy to accidentally cut during anterior vitrectomy.

The safety of the procedure needs to be taken care of here: the negative pressure suction of the vitreous cutter needs to be gentle. Specifically, the vitreous cutter opening faces up and initiates the negative pressure suction mode close to the posterior capsule of the lens. When the anterior vitreous cortex is about to be completely excised, the entire lens capsular flutters and converges toward the vitreous cutter. Subsequently, radial folds of the posterior capsular of the lens indicate complete resection of the anterior vitreous body. After the surgeon and his assistant jointly confirmed that according to this method in all 26 patients in the experimental group, no posterior capsule of the lens was ruptured.

This study focuses on introducing a method of locating the anterior vitreous cortex based on intraoperative observations. Additionally, the vitreous body cannot be specifically quantified by current ophthalmic examination methods, including intraoperative OCT, anterior segment OCT, ophthalmic B-ultrasound, and ocular ultrasonic biomicroscopy. In this study, we could only use triamcinolone to determine whether the vitreous cortex remained. The results showed that 2 patients in each group still had residual vitreous anterior cortex, and there was no significant difference.

The limitation of this study is that the quantity of the vitreous body depends on the subjective judgment of the surgeon. Further research will need to explore more objective and quantitative indicators.

## Data Availability

All data used and analyzed in this study are available upon request from the first author: Shen Qu.
